# Maternal fish oil supplementation improves metabolic and inflammatory markers in mice overfed during the postnatal period

**DOI:** 10.3389/fnut.2025.1685437

**Published:** 2025-11-17

**Authors:** Isabela Queiroz Perígolo Lopes, Brenda Loise Monteiro, Adaliene Versiani Matos Ferreira, Rodrigo Ferreira de Moura, Janina de Sales Guilarducci, Estéfany Ribeiro Leão, Bárbara do Carmo Rodrigues Virote, Isaac Konig, Luis David Solis Murgas, Isabela Coelho de Castro, Laura Cristina Jardim Porto Pimenta

**Affiliations:** 1 Department of Nutrition, Federal University of Lavras, Lavras, Brazil; 2 Department of Nutrition, Federal University of Minas Gerais, Belo Horizonte, Brazil; 3 Department of Medicine, Federal University of Lavras, Lavras, Brazil; 4 Department of Agriculture, Federal University of Lavras, Lavras, Brazil; 5 Department of Veterinary Medicine, Federal University of Lavras, Lavras, Brazil; 6 Department of Chemistry, Federal University of Mato Grosso, Cuiabá, Brazil

**Keywords:** metabolic programming, overfeeding, omega-3, polyunsaturated fatty acids, oxidative stress, obesity

## Abstract

**Background:**

Early-life nutrition, especially during gestation and lactation, plays a key role in metabolic programming and can influence the risk of obesity and related conditions in adulthood. This study investigated whether supplementation with maternal fish oil—rich in omega-3 polyunsaturated fatty acids—could prevent metabolic and inflammatory changes induced by postnatal overfeeding.

**Methods:**

Female mice received fish oil (1 g/kg by oral gavage) during mating, pregnancy, and lactation. The animals were allocated into three groups: control (C), postnatal overfeeding (PO), and postnatal overfeeding + fish oil (POFO). Neonatal overfeeding was induced by reducing litter size, and only male offspring were analyzed. In adulthood, body weight, glucose tolerance, lipid profile, serum adipokines, adipose tissue cytokines, and hepatic oxidative stress markers were evaluated.

**Results:**

Maternal fish oil supplementation reduced early weight gain and lowered fasting glucose, total cholesterol, and low-density lipoprotein (LDL) levels, while increasing high-density lipoprotein (HDL) levels in overfed offspring. It also decreased serum leptin, resistin, and chemerin levels and reduced hepatic lipid peroxidation, thereby restoring catalase activity. No differences were observed in hepatic triglycerides or superoxide dismutase activity.

**Conclusion:**

Maternal fish oil supplementation during critical developmental windows attenuated the metabolic, inflammatory, and oxidative stress alterations induced by postnatal overfeeding in male mice.

## 1 Introduction

Childhood obesity has become a global public health problem, with its prevalence and severity continuing to rise among pediatric populations worldwide. It can affect multiple organs and is associated with significant morbidity and premature mortality, with complications such as dyslipidemia, hypertension, fatty liver disease, and psychosocial issues (1–3). Several public health interventions are currently available to prevent childhood obesity, including federal nutrition assistance initiatives, early childhood education initiatives, school-based interventions, community programs, food labeling and marketing regulations, and taxes on sugar-sweetened beverages (3). However, it is also important to note that inadequate parental eating habits can enhance the probability of developing childhood obesity (4, 5). In this sense, metabolic programming stands out as a strategy for preventing obesity and being overweight from the earliest stages of life (6).

A balanced diet is important from the intrauterine period because, in the first years of life, the individual is exposed to metabolic programming that can modulate the risk of obesity and other chronic diseases in adulthood (7). This concept is referred to as the developmental origins of health and disease (DOHaD) and aims to understand how early life events affect later life outcomes, especially the development of non-communicable chronic diseases (8). Postnatal overfeeding models may be a predisposing factor for metabolic disorders in adulthood (9).

The litter size reduction model in rodents is an efficient experimental tool for investigating the effects of postnatal overnutrition on metabolic disorder outcomes. In rodents, dams usually produce 10–12 pups; therefore, reducing the number of pups on the third day of lactation reduces competition and increases the availability of food for the offspring (10, 11). Therefore, this model is particularly suitable for investigating the long-term effects of fish oil (FO) administration on the metabolic, inflammatory, and oxidative status of offspring during adulthood.

Among various dietary interventions for metabolic programming strategies, supplementation with omega-3 (*ω*-3) polyunsaturated fatty acids (PUFAs) has garnered particular attention. These fatty acids have several beneficial effects on metabolic and inflammatory pathways, including a reduction in plasma lipid concentration, improved membrane fluidity, enhanced signal transduction and gene expression, improved immune function, and enhanced insulin signaling (11–13). It is well known that *ω*-3 s, such as eicosapentaenoic acid (EPA) and docosahexaenoic acid (DHA), can bind to peroxisome proliferator-activated receptor alpha (PPARα) and act as primary regulators of transcriptional events (11) and may play a key role in metabolic programming events through epigenetic modifications.

Fish oil (FO) is composed of essential *ω*-3 PUFAs, primarily EPA and DHA (14). FO supplementation during pregnancy and postpartum in mothers with overweight and obesity has been associated with a 17 and 21% reduction in maternal and infant triglycerides, respectively, without affecting infant body composition (15). On the other hand, paternal FO supplementation has been associated with lower body weight, improved insulin responsiveness, and modifications in inflammatory markers in the male progeny (16, 17). Nevertheless, the effects of FO supplementation, specifically during the gestation and lactation periods, on metabolic, inflammatory, and oxidative status require further investigation.

In this context, thoroughly investigating the metabolic effects of fish oil as a strategy to mitigate obesity and related conditions is highly relevant. Therefore, this study aimed to evaluate the impact of maternal fish oil supplementation from gestation through lactation on metabolic and inflammatory markers in mice subjected to postnatal overfeeding.

## 2 Materials and methods

### 2.1 Animal studies

All procedures involving animals were approved by the local Institutional Animal Care and Use Committee (CEUA/002/2018). C57Bl/6JUnib mice were obtained from the Federal University of Minas Gerais (UFMG, Belo Horizonte, Brazil).

### 2.2 Experimental design

**Figure 1:**
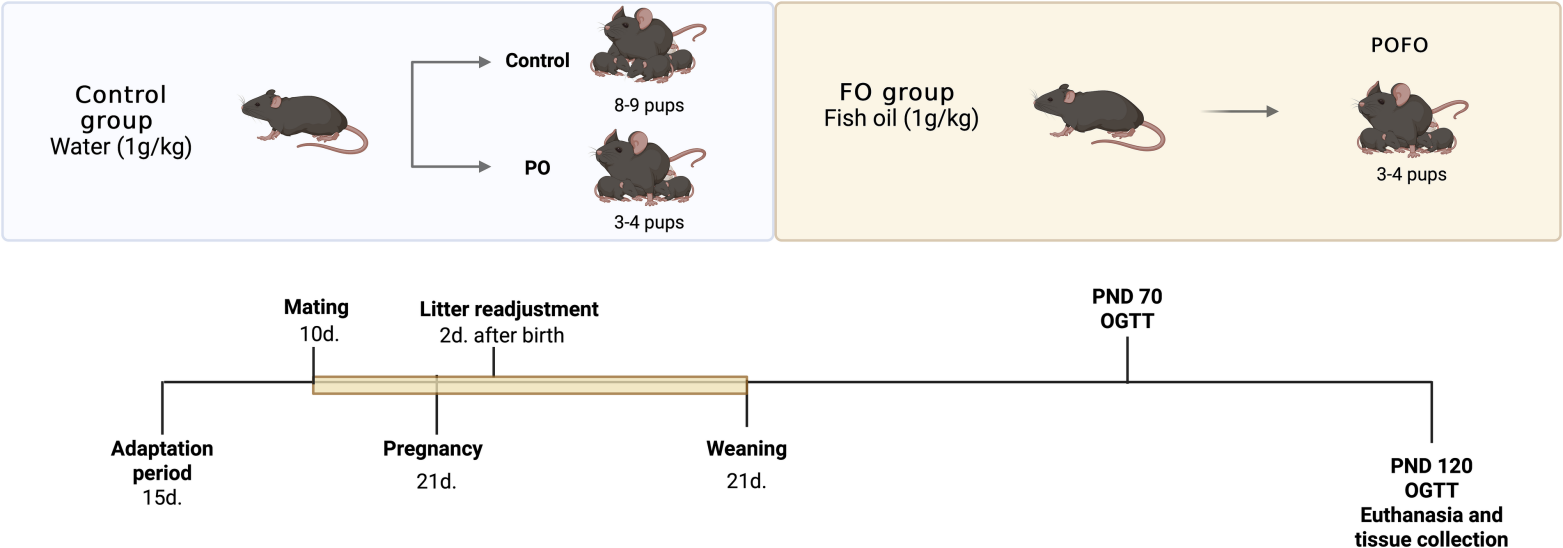
Experimental design. Schematic representation of the experimental protocol. From mating until the end of lactation, the mothers received either water (Control group, 1 g/kg) or fish oil (FO group, 1 g/kg) by oral gavage. Two days after birth, litter size was adjusted to 8–9 pups per dam (Control) or 3–4 pups per dam to induce postnatal overfeeding (PO and POFO groups). Offspring were weaned on postnatal day (PND) 21. Glucose tolerance tests (OGTT) were performed at PND 70 and PND 120, followed by euthanasia and tissue collection at PND 120.

Forty C57Bl/6 J mice (10 males and 30 females), aged 45–55 days and weighing approximately 20 g, were obtained from the animal care facility at the Federal University of Lavras. The animals were maintained under standard conditions, following institutional ethical guidelines, with free access to water and standard chow, composed of 44.5% carbohydrates, 23% protein, 4.5% ether extract, 5% fiber, 10% ash, and 13% moisture. The experimental design is illustrated in Figure 1.

Female mice were housed in groups of three animals per cage for a 15-day adaptation period. The temperature was maintained at 22 °C with a 12-h light/dark cycle to simulate natural environmental conditions. Following this, one male was introduced to each group of three females for a 10-day mating period. After mating, the males were removed, and the females remained together until parturition.

The experimental groups were predetermined to induce postnatal overfeeding; litter size was adjusted to 3–4 male pups per dam 2 days after birth to form the postnatal overfeeding (PO) and postnatal overfeeding + fish oil supplementation (POFO) groups, according to the protocol described by Habbout et al. (18). Litters with 8–10 pups per dam were used as the control group. The animals remained in these group formations until weaning, and only the male offspring were used in the study to avoid the potential confounding effects of hormonal fluctuations during the estrous cycle, as they can influence metabolic and inflammatory responses. The dams received daily oral gavage with either fish oil (FO) or water (1 g/kg body weight) from the start of mating until weaning (21 days postpartum), totaling approximately 34 days of supplementation. From weaning until 120 days of age, the offspring had free access to water and a standard diet *ad libitum*. The FO dosage was selected according to previous studies (19, 20).

The FO used was a marine lipid preparation rich in *ω*-3 fatty acids (Herbarium Foundation®/Curitiba, PR, Brazil) containing 0.828 g per capsule, with 0.120 g of EPA and 0.300 g of DHA. Body weight and food intake were monitored weekly.

One day after reaching 120 days of age, the animals were fasted for 12 h and subsequently anesthetized via intraperitoneal injection of a ketamine (150 mg/kg) and xylazine (10 mg/kg) mixture. Euthanasia was carried out by cardiac exsanguination. The liver, epididymal adipose tissue, and blood were collected. All tissues were weighed immediately after euthanasia. Blood samples were centrifuged at 1,500 rpm for 10 min using a CentriBio® (Curitiba, PR, Brazil) centrifuge to obtain serum. Both serum and tissue samples were stored at −20 °C until analysis.

### 2.3 Oral glucose tolerance test

Oral glucose tolerance tests (OGTT) were conducted in mice at 70 and 120 days of age. Day 70 was selected to evaluate intermediate outcomes during early adulthood, while day 120 was used to assess outcomes in established adulthood. Following a 12-h overnight fast with free access to water. Blood samples were collected from the tail vein at 0, 30, 60, 90, and 120 min after oral glucose administration by gavage (2 mg/g body weight). Glucose levels were determined using an Accu-Chek glucometer (Roche Diagnostics, Indianapolis, IN, USA) and expressed in mmol/L. The area under the curve (AUC) was determined using the trapezoidal method based on glucose concentrations over time.

### 2.4 Determination of metabolic markers

Fasting blood glucose, triacylglycerol, total cholesterol, and HDL-cholesterol levels were measured using colorimetric kits (Labtest, Lagoa Santa, MG, Brazil), following the manufacturer’s instructions. The LDL-c fraction was calculated using the Friedewald formula: LDL-c = [(triglycerides/5) + HDL-c] − total cholesterol (21). Serum levels of adiponectin, leptin, chemerin, and resistin were quantified by Enzyme-Linked Immunosorbent Assay (ELISA) using kits from R&D Systems Europe Ltd (Abingdon, UK), following the manufacturer’s protocols.

### 2.5 Determination of inflammatory markers in adipose tissue

Tumor necrosis factor-*α* (TNF-α) and interleukin-10 (IL-10) levels were measured in epididymal adipose tissue using DuoSet ELISA development kits (R&D Systems, Inc., Minneapolis, MN, USA), following the manufacturer’s instructions.

### 2.6 Determination of hepatic lipids

Total hepatic lipids were extracted using organic solvents, following the method described by Folch et al. (22). The lipid extracts were dried overnight at 37 °C and subsequently quantified. Concentrations of total cholesterol and triglycerides were determined using commercial kits (Labtest, Lagoa Santa, MG, Brazil) in lipid extracts diluted in 500 μL of isopropanol.

### 2.7 Oxidative stress in the liver

Liver samples (100 mg) were homogenized in phosphate-buffered saline (PBS) using a mechanical homogenizer. After centrifugation, the homogenates were stored at −20 °C until analysis. Lipid peroxidation was assessed by measuring thiobarbituric acid reactive substances (TBARS), as described by Wallin et al. (23), with results expressed as nmol of MDA/mg of protein. Hydroperoxide concentrations were determined according to the method described by Banerjee et al. (24). Superoxide dismutase (SOD) activity was measured by assessing the inhibition of pyrogallol auto-oxidation and monitoring the absorbance at 550 nm (25). Catalase (CAT) activity was evaluated by measuring the rate of decomposition of hydrogen peroxide (H₂O₂) at 240 nm, following Aebi’s method (26). The total protein content was determined using the Bradford method (27) and was used to normalize all oxidative stress parameters.

### 2.8 Statistical analysis

The results are presented as the mean ± standard deviation. Graphing and statistical analyses were conducted using GraphPad Prism 9.0 (GraphPad Software, La Jolla, CA, USA). Data normality was assessed using the Shapiro–Wilk test. Possible outliers were identified and removed based on Grubbs’ test. For comparisons between two groups, Student’s *t*-test was applied, while a one-way analysis of variance (ANOVA) followed by Bonferroni’s *post-hoc* test was used for multiple group comparisons. Statistical significance was set at a *p*-value of < 0.05.

## 3 Results

### 3.1 Body parameters

Body weight gain and organ weights are summarized in Table 1. At 21 days of age, the postnatally overfed (PO) group exhibited significantly higher body weight compared to the control group (C) (*p* < 0.001). By 120 days, body weight remained elevated in the PO group compared to the control group (*p* < 0.01).

**Table 1:** Body weight (g) and liver and epididymal adipose tissue weights of experimental groups.

Parameters	C	PO	POFO	PO vs. C	POFO vs. PO
BW at 21 days (g)	8.2 ± 0.2	10.2 ± 0.4	9.0 ± 0.2	*p* < 0.001	ns
BW at 120 days (g)	24.7 ± 0.2	26.1 ± 0.3	25.4 ± 0.6	*p* < 0.01	ns
Liver (mg/g BW)	41.8 ± 1.7	46.5 ± 1.3	44.9 ± 1.3	*p* < 0.05	*p* < 0.05
Epididymal adipose tissue (mg/g BW)	8.9 ± 0.2	14.3 ± 0.7	13.0 ± 0.4	*p* < 0.0001	ns

BW, Body weight; ns: no significant. Control (C, *n* = 8), Postnatal Overfeeding (PO, *n* = 8), and Postnatal Overfeeding + Fish Oil supplementation (POFO, *n* = 14). Data are presented as the mean ± standard error of the mean.

The liver weight normalized by body weight was significantly higher in the PO group compared to the control group (*p* < 0.05) and was lower in the POFO group compared to the PO group (p < 0.05). Additionally, the epididymal adipose tissue weight normalized by body weight was markedly increased in the PO group compared to the control group (*p* < 0.0001), with no significant difference between the PO and POFO groups (*p* > 0.05).

### 3.2 Metabolic markers

Blood serum analysis showed elevated fasting glucose levels in the PO group compared to the control group, while the POFO group had lower levels than the PO group (*p* < 0.05). Maternal fish oil supplementation also improved total cholesterol and LDL-c levels in the POFO group compared to the PO group (*p* < 0.0001), both of which were elevated in the PO group relative to the C group (p < 0.0001), and elevated HDL-c levels in the POFO group than in the PO group (*p* < 0.05). Triglyceride levels and total hepatic lipid and cholesterol content did not differ among the groups (*p* > 0.05). However, hepatic triglyceride levels were increased in the PO group compared to the control group (*p* < 0.01), with no significant differences observed between the POFO group and PO group (*p* > 0.05) (Table 2).

**Table 2:** Serum and hepatic metabolic markers of the experimental groups.

Parameters	C	PO	POFO	PO vs. C	POFO vs. PO
Blood serum					
Fasting glucose (mmol/L)	6.5 ± 0.3	8.2 ± 0.2*	6.6 ± 0.3	*p* < 0.05	*p* < 0.05
Triglycerides (mmol/L)	0.5 ± 0.1	0.5 ± 0.1	0.6 ± 0.1	ns	ns
Total cholesterol (mmol/L)	1.9 ± 0.1	3.7 ± 0.2	2.3 ± 0.2	*p* < 0.0001	*p* < 0.0001
LDL-c (mmol/L)	0.6 ± 0.1	2.0 ± 0.2	0.6 ± 0.1	*p* < 0.0001	*p* < 0.0001
HLD-c (mmol/L)	1.3 ± 0.1	1.1 ± 0.1	1.6 ± 0.1	ns	*p* < 0.05
Hepatic lipids					
Total lipids (mg of lipids/g of liver)	3.4 ± 0.2	3.2 ± 0.2	4.1 ± 0.4	ns	ns
Cholesterol (mmol/L)	5.0 ± 0.3	5.5 ± 0.3	4.4 ± 0.5	ns	ns
Triglycerides (mmol/L)	2.8 ± 0.3	4.6 ± 0.1	3.3 ± 0.5	*p* < 0.01	ns

ns, not significant.

Control (C, *n* = 8), Postnatal Overfeeding (PO, *n* = 8), and Postnatal Overfeeding + Fish Oil supplementation (POFO, *n* = 14). Data are presented as the mean ± standard error of the mean.

**Figure 2:**
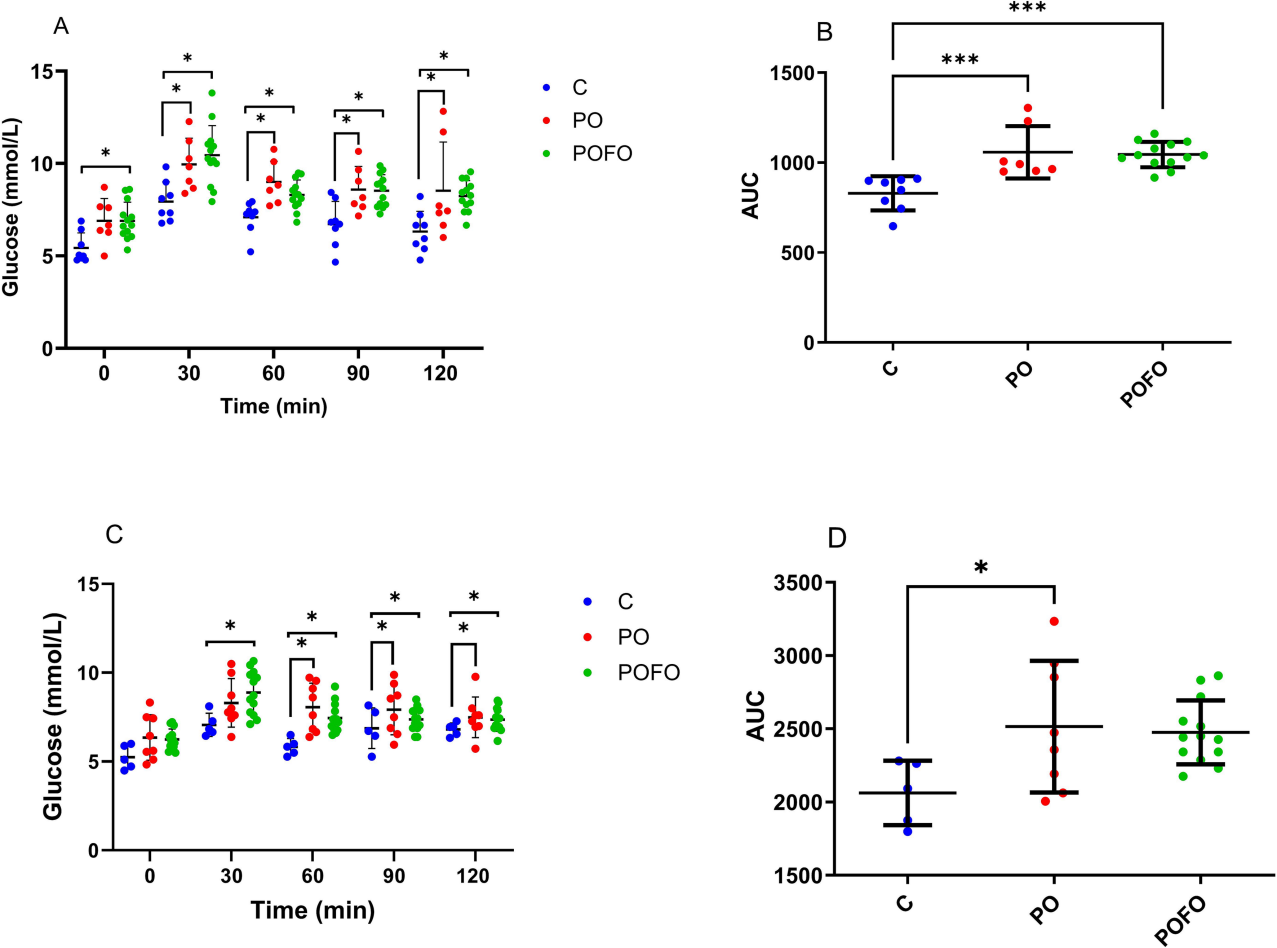
Glycemic curve (mmol/L) versus time (min) from the oral glucose tolerance test (OGTT) and the Area Under the Curve at 70 **(A,B)** and 120 **(C,D)** days for male pups. Control (C, *n* = 5–8), Postnatal Overfeeding (PO, *n* = 7–8), and Postnatal Overfeeding + Fish Oil supplementation (POFO, *n* = 13). Data are presented as the mean ± standard error. At 70 days, both the POFO and PO groups exhibited higher blood glucose levels at 0, 30, 60, 90, and 120 min compared to the control group (*p* < 0.05), whereas at 120 days, only at 0 min there were no differences between the control and treatment groups **(C)**. The AUC at 120 days showed higher glucose levels in the PO group compared to the control group, whereas the POFO group showed no significant difference **(D)**. *p < 0.05 vs. the Control group; ***p < 0.001 vs. the Control group.

To further assess glucose tolerance, oral glucose tolerance tests (OGTT) were conducted at 70 and 120 days of age. At 70 days, the PO group exhibited a higher glycemic curve compared to the control group (*p* < 0.05). The POFO group showed no difference relative to the PO group in the AUC curve (Figures 2A,B). At 70 days, both the POFO and PO groups exhibited higher blood glucose levels at 0, 30, 60, 90, and 120 min compared to the control group (*p*<0.05), whereas at 120 days, only at 0 min there were no differences between the control and treatment groups (Figure 2C). The AUC at 120 days showed higher glucose levels in the PO group compared to the control group, whereas the POFO group showed no significant difference (Figure 2D).

**Figure 3:**
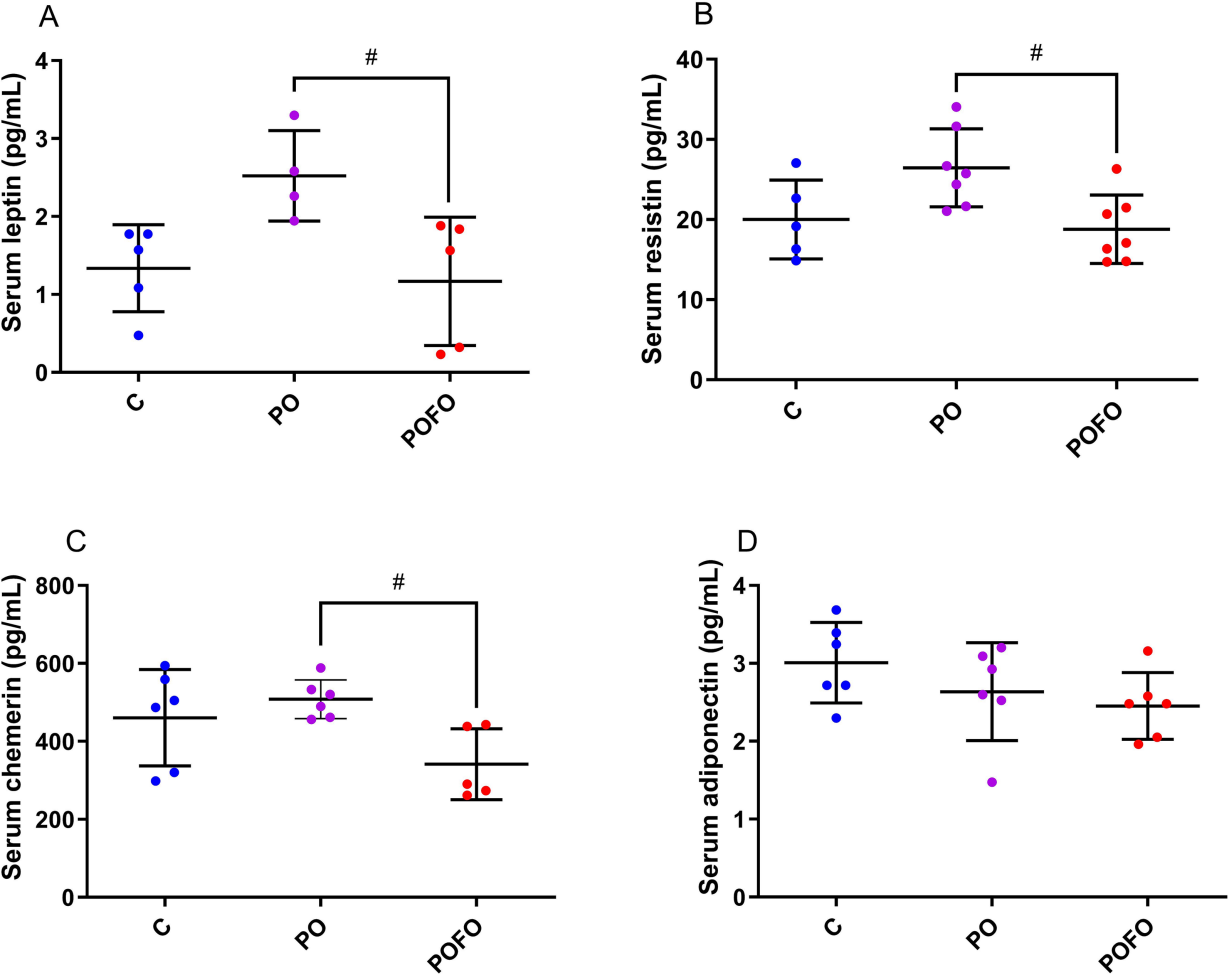
Leptin **(A)**, resistin **(B)**, chemerin **(C)**, and adiponectin **(D)** levels in the serum of male pups at 120 days. Control (C, *n* = 5–6), Postnatal Overfeeding (PO, *n* = 4–7), and Postnatal Overfeeding + Fish Oil supplementation (POFO, *n* = 5–7). Data are presented as the mean ± standard error. #*p* < 0.05 vs. the PO group.

Serum leptin and resistin levels were elevated in the PO group compared to the control group (*p* < 0.05 for both), while the POFO group showed reduced levels compared to the PO group (*p* < 0.05 for both) (Figures 3A,B). Chemerin levels were also lower in the POFO group compared to the PO group (*p* < 0.05) (Figure 3C). No significant differences in serum adiponectin levels were observed among the groups (*p* > 0.05) (Figure 3D).

### 3.3 Inflammatory responses

**Figure 4:**
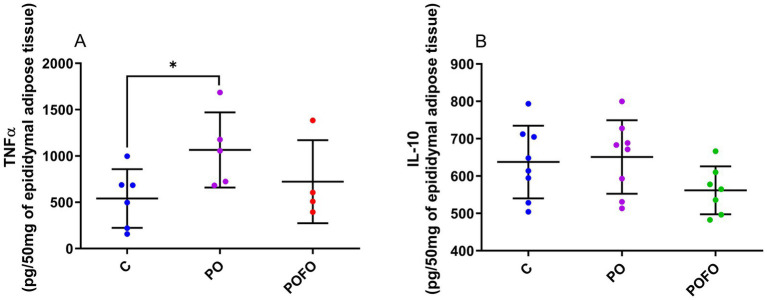
TNF-α **(A)** and IL-10 **(B)** levels in the epididymal adipose tissue of male pups at 120 days. Control (C, *n* = 6–8), Postnatal Overfeeding (PO, *n* = 5–8), and Postnatal Overfeeding + Fish Oil supplementation (POFO, *n* = 4–7). Data are presented as the mean ± standard error. *p < 0.05 vs. the Control group.

Analysis of the inflammatory profile in epididymal adipose tissue revealed elevated TNF-*α* levels in the PO group compared to the control group (*p* < 0.05), whereas such an elevation was not observed in the POFO group (Figure 4A). In contrast, levels of the anti-inflammatory cytokine IL-10 did not differ significantly among the groups (*p* > 0.05) (Figure 4B).

### 3.4 Oxidative stress

**Figure 5:**
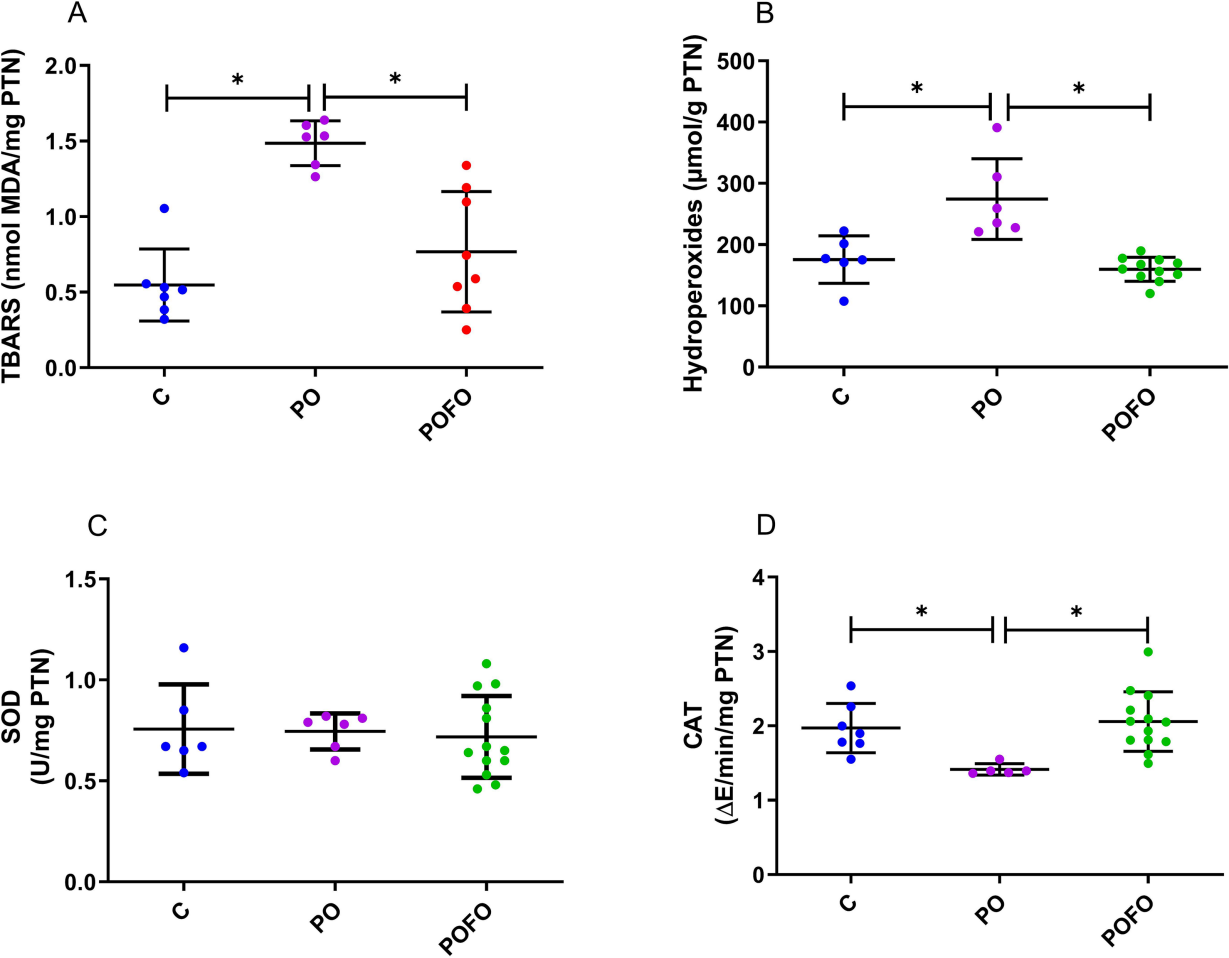
Oxidative stress in the livers of male pups at 120 days. Determination of thiobarbituric acid reactive substances (TBARS) according to the concentration of malondialdehyde (MDA) **(A)**, hydroperoxides according to the concentration of hydroperoxides **(B)**, and the activity of the antioxidant enzymes superoxide dismutase (SOD) **(C)** and catalase (CAT) **(D)**, normalized by the protein concentration in the livers of the animals. Control (C, *n* = 6–7), Postnatal Overfeeding (PO, *n* = 5–6), and Postnatal Overfeeding + Fish Oil supplementation (POFO, *n* = 8–13). Data are presented as the mean ± standard error. *p < 0.05.

Analysis of hepatic oxidative stress markers revealed that postnatal overfeeding significantly increased MDA levels (Figure 5A) and hydroperoxide levels (Figure 5B) (*p* < 0.0001 and *p* < 0.01, respectively). At the same time, maternal fish oil supplementation significantly reduced both parameters (*p* < 0.001 vs. PO). No significant differences in SOD activity were observed among the groups (*p* > 0.05) (Figure 5C). However, CAT activity was reduced in the PO group compared to the control group (*p* < 0.01), and maternal fish oil supplementation induced an increase in this parameter compared to the PO group (*p* < 0.05) (Figure 5D).

## 4 Discussion

This study demonstrates that maternal supplementation with fish oil (FO) during critical developmental periods confers sustained protective effects on male offspring exposed to postnatal overfeeding, observed up to 120 days of age. Notably, FO was able to attenuate early excessive weight gain and modulate key inflammatory and oxidative stress markers. A novel contribution of our findings is that maternal FO intake during lactation, rather than direct supplementation to the offspring, appears to attenuate metabolic and inflammatory dysfunctions associated with neonatal overnutrition, highlighting the potential importance of maternal nutritional interventions in developmental programming.

Postnatal overfeeding, induced by small litter size, leads to a chronic low-grade inflammatory state, evidenced by increased epididymal fat mass and elevated serum levels of leptin and resistin, along with higher TNF-*α* levels in adipose tissue (28–31). These alterations are associated with adipocyte hypertrophy and hypoxia, which stimulate excessive adipokine release and contribute to systemic inflammation. Leptin, in particular, plays a central role in energy homeostasis by promoting fatty acid oxidation; however, in obesity, hyperleptinemia often reflects leptin resistance, a condition that amplifies insulin resistance (32). Resistin is another adipokine implicated in the pathophysiology of insulin resistance and is found at elevated levels in individuals with a higher BMI, showing strong positive correlations with leptin and insulin concentrations, thereby reinforcing its involvement in glucose dysregulation (33).

The rise in adipokines was accompanied by increased lipid peroxidation and oxidative stress. These effects may result from enhanced mitochondrial reactive oxygen species (ROS) production due to metabolic overload and inflammatory signaling. Markers such as malondialdehyde (MDA) and oxidized LDL (Ox-LDL) are significantly elevated in obesity, indicating sustained oxidative damage to lipids and vascular tissues, which further impairs insulin signaling (34). These alterations likely contributed to the glucose intolerance observed at both 70 and 120 days, suggesting decreased insulin sensitivity, consistent with the findings of Bei et al. (35). The presence of systemic and tissue-specific oxidative imbalance, reduced antioxidant capacity, and chronic low-grade inflammation was similarly demonstrated in those studies, further confirming the long-term metabolic consequences of neonatal overnutrition (10, 36).

Conversely, maternal FO supplementation prevented early weight gain, and although final body weight and adipose tissue were unchanged, leptin, resistin, and chemerin levels were restored in the offspring. Similar outcomes were reported by other studies that supplemented *ω*-3 s during gestation and lactation (30, 31). Excess body weight and insulin resistance can impair leptin signaling, contributing to leptin resistance. Emerging evidence suggests that *ω*-3 fatty acids can alleviate leptin resistance and modulate the expression of adipokine genes (35). This effect is partially explained by the ability of long-chain omega-3 polyunsaturated fatty acids to reduce systemic inflammation and improve leptin signaling pathways, such as in Refs. (37, 38). These fatty acids can also modulate the expression of genes related to lipid metabolism and adipogenesis, such as PPAR-*γ* and SREBP-1c, decrease leptin mRNA expression in white adipose tissue, and enhance cellular membrane composition, factors that together contribute to improved leptin sensitivity and reduced circulating leptin levels (39, 40). Maternal metabolism is a key factor in determining the availability and abundance of nutrients supplied to the fetus. This nourishment is dynamically regulated throughout pregnancy to support fetal development (10). The fatty acid composition delivered to the fetus is largely determined by maternal circulating levels. Long-chain polyunsaturated fatty acids are preferentially transferred across the placenta due to their physiological importance (36).

A recent meta-analysis reported that direct supplementation in humans with doses ≥ 2 g/day of EPA + DHA may significantly reduce circulating leptin levels (41). Previous reports have shown that maternal supplementation with fish oil (FO) improves the levels of EPA and DHA in breast milk (42). Furthermore, omega-3 PUFAs have been shown to inhibit the secretion of chemerin from adipocytes via G-protein–coupled receptor 120 signaling, attenuating its pro-inflammatory effects (43). Resistin, an adipokine associated with insulin resistance and inflammation, has been found to decrease after omega-3 supplementation in rodent and human tissues, although results are variable depending on the dose and model (44).

Specifically, the adipokine chemerin has gained attention as a critical mediator linking adipose tissue dysfunction to systemic metabolic and inflammatory disturbances. Chemerin is closely associated with obesity, inflammation, and vascular dysfunction, acting as a key regulator of glucose and lipid metabolism through the modulation of insulin sensitivity, lipolysis, and adipocyte differentiation (45, 46). The nutritional modulation of the chemerin/ChemR23 axis has gained attention due to the actions of omega-3 polyunsaturated fatty acids, particularly EPA, which can give rise to lipid mediators such as resolvin E1, which binds to ChemR23 and exerts anti-inflammatory and insulin-sensitizing effects (47). Furthermore, Tan et al. (43) demonstrated in their review that diets enriched with omega-3 fatty acids can suppress systemic chemerin expression, whereas obesogenic diets tend to upregulate this adipokine, reinforcing the relevance of dietary quality in modulating metabolic inflammation.

In our study, lower fasting glycemia was observed in fish oil-treated mice, whereas glucose tolerance was unchanged at 70 and 120 days. Albert et al. (48) also reported no improvement in glucose tolerance in DHA- and EPA-supplemented Sprague–Dawley rats. Omega-3 fatty acids have been implicated in the regulation of the insulin signaling pathway, including PKC and GLUT4, which may be influenced by maternal diet and impact glucose uptake in adult offspring (49). Research on the long-term impact of fish oil consumption during fetal development or early life on the glucose-insulin system in animals is limited and inconsistent, particularly in rodent studies. These findings indicate that the dosage and timing of supplementation play a crucial role (50). Future studies measuring plasma insulin and investigating hepatic and muscle insulin signaling (PEPCK/G6Pase, p-AKT, and GLUT4) are warranted to clarify the underlying mechanisms.

Maternal FO supplementation improved lipid profiles in overfed offspring by reducing total serum cholesterol and LDL-c, and increasing HDL-c. These changes are consistent with previous studies on the effects of omega-3 s in the maternal diet (51, 52), though Kasbi-Chadli et al. (51) reported no differences in VLDL, LDL, or HDL. Omega-3 s are known to enhance hepatic LDL receptor expression and cholesterol excretion via bile acids (53). However, our study did not detect FO effects on hepatic lipid accumulation, differing from the findings of Sánchez-Blanco et al. (54), who reported lower hepatic lipids in the offspring of FO-supplemented mothers fed a cafeteria diet. It is important to highlight that fish oil supplementation during the gestational period of Wistar rats decreased the hepatic expression of lipogenic genes and reduced hepatic mitochondrial damage in both male and female offspring (55).

Another protective effect of maternal fish oil supplementation was observed in the liver, specifically with regard to oxidative stress-related damage. These results suggest that maternal nutritional intervention with fish oil was able to attenuate hepatic oxidative damage induced by postnatal overfeeding. Malondialdehyde (MDA) is one of the most commonly used biomarkers for assessing oxidative stress, as it is a stable product of lipid peroxidation derived from polyunsaturated fatty acids (PUFAs), particularly arachidonic acid (56). Elevated MDA levels, typically measured using the TBARS assay in plasma or serum, have been associated with various diseases characterized by enhanced lipid peroxidation (57). Catalase is a peroxidase enzyme responsible for degrading hydrogen peroxide (H₂O₂) into water and oxygen, thereby neutralizing its cytotoxic effects and playing a key role in cellular antioxidant defense 55. In contrast to our findings, Kasbi-Chadli et al. (51) did not observe any significant effects of maternal fish oil supplementation on MDA levels, glutathione peroxidase activity, or plasma superoxide dismutase (SOD) in offspring from dams fed a cafeteria diet. However, in a different experimental model, Miyaguti, Oliveira, and Gomes-Marcondes (58) reported that maternal fish oil supplementation was able to mitigate hepatic oxidative stress in tumor-bearing rats, reinforcing its potential antioxidant role in pathological conditions.

The mechanism by which *ω*-3 indirect supplementation leads to an improvement in metabolic and oxidative stress and inflammatory markers may be related to metabolic/epigenetic programming effects during early life. Further studies are required to address the effects of maternal supplementation with FO on gene expression and epigenetic mechanisms.

This study has some limitations, including the exclusive use of male offspring, the absence of fatty acid profile measurements to confirm ω-3 status, and the lack of detailed characterization of the fish oil used. The small litter size model may also involve behavioral factors beyond nutrition. We recommend that future studies incorporate a control + fish oil group to evaluate the effects of supplementation in non-overfed offspring. Mechanistic explanations remain speculative, as no molecular or epigenetic analyses were performed. Finally, the translation of these findings to humans requires caution due to physiological and dietary differences.

## 5 Conclusion

Maternal fish oil supplementation during critical developmental periods attenuated the metabolic, inflammatory, and oxidative stress alterations induced by postnatal overfeeding in male mice. These benefits included improved glucose tolerance, modulation of adipokine profiles, and protection against hepatic oxidative damage, reinforcing the importance of maternal nutrition in shaping long-term health outcomes in the offspring. Although the precise mechanisms remain to be elucidated, these findings support the potential role of *ω*-3 fatty acids in early-life nutritional programming. Future studies should further explore the metabolic implications of maternal fish oil supplementation, for example, through the quantification of insulin and related markers of glucose homeostasis. Additionally, these studies should include both sexes, assess molecular and epigenetic pathways, and explore the translational applicability of these results to human populations.

## Data Availability

The original contributions presented in the study are included in the article/supplementary material, further inquiries can be directed to the corresponding author.
